# Supplementary material for: Resolving the full spectrum of human genome variation using Linked-Reads
Source: Genome Res. 2019 Apr;29(4):635–45. doi: 10.1101/gr.234443.118 (PMC6442396; doi:10.1101/gr.234443.118)
Supplement: Supplemental Material [file supp_gr.234443.118_Supplemental_longranger-2.2.zip › longranger-2.2.tar.bz2!/lr2.2-public/lib/stan/cmdstan/stan/doc/api/html/functions_o.html]

Stan: Class Members


|  |  |
| --- | --- |
|  | Stan  2.5.0  probability, sampling & optimization |


- Main Page
- Related Pages
- Namespaces
- Classes
- Files

- Class List
- Class Index
- Class Hierarchy
- Class Members

- All
- Functions
- Variables
- Typedefs
- Enumerator
- Related Functions

- \_
- a
- b
- c
- d
- e
- f
- g
- h
- i
- j
- k
- l
- m
- n
- o
- p
- q
- r
- s
- t
- u
- v
- w
- x
- y
- z
- ~


All Classes Namespaces Files Functions Variables Typedefs Enumerations Enumerator Friends Macros Pages

Here is a list of all class members with links to the classes they belong to:

### - o -

- o
  : stan::model::model\_functional< M >
- o\_
  : stan::gm::visgen
- OK
  : stan::gm::error\_codes
- op
  : stan::gm::binary\_op
  , stan::gm::unary\_op
- op\_ddv\_vari()
  : stan::agrad::op\_ddv\_vari
- op\_dv\_vari()
  : stan::agrad::op\_dv\_vari
- op\_dvd\_vari()
  : stan::agrad::op\_dvd\_vari
- op\_dvv\_vari()
  : stan::agrad::op\_dvv\_vari
- op\_matrix\_vari()
  : stan::agrad::op\_matrix\_vari
- op\_v\_vari()
  : stan::agrad::op\_v\_vari
- op\_vd\_vari()
  : stan::agrad::op\_vd\_vari
- op\_vdd\_vari()
  : stan::agrad::op\_vdd\_vari
- op\_vdv\_vari()
  : stan::agrad::op\_vdv\_vari
- op\_vector\_vari()
  : stan::agrad::op\_vector\_vari
- op\_vv\_vari()
  : stan::agrad::op\_vv\_vari
- op\_vvd\_vari()
  : stan::agrad::op\_vvd\_vari
- op\_vvv\_vari()
  : stan::agrad::op\_vvv\_vari
- OperandsAndPartials()
  : stan::agrad::OperandsAndPartials< T1, T2, T3, T4, T5, T6, T\_return\_type >
- operator delete()
  : stan::agrad::chainable
- operator new()
  : stan::agrad::chainable
- operator!=()
  : stan::gm::expr\_type
- operator()()
  : add\_var
  , copy\_square\_cholesky\_dimension\_if\_necessary
  , data\_only\_expression
  , empty\_range
  , set\_double\_range\_lower
  , set\_double\_range\_upper
  , set\_int\_range\_lower
  , set\_int\_range\_upper
  , stan::common::dump\_factory
  , stan::common::NoOpFunctor
  , stan::common::recorder::csv
  , stan::common::recorder::filtered\_values< InternalVector >
  , stan::common::recorder::messages
  , stan::common::recorder::no\_op
  , stan::common::recorder::sum\_values
  , stan::common::recorder::values< InternalVector >
  , stan::common::var\_context\_factory< VARCON >
  , stan::gm::add\_conditional\_body
  , stan::gm::add\_conditional\_condition
  , stan::gm::add\_expression\_dimss
  , stan::gm::add\_fun\_var
  , stan::gm::add\_function\_signature
  , stan::gm::add\_loop\_identifier
  , stan::gm::add\_while\_body
  , stan::gm::add\_while\_condition
  , stan::gm::addition\_expr3
  , stan::gm::binary\_op\_expr
  , stan::gm::constrained\_param\_names\_visgen
  , stan::gm::contains\_nonparam\_var
  , stan::gm::contains\_var
  , stan::gm::division\_expr
  , stan::gm::dump\_member\_var\_visgen
  , stan::gm::elt\_division\_expr
  , stan::gm::elt\_multiplication\_expr
  , stan::gm::exponentiation\_expr
  , stan::gm::expression\_as\_statement
  , stan::gm::expression\_type\_vis
  , stan::gm::expression\_visgen
  , stan::gm::generate\_init\_vars\_visgen
  , stan::gm::generate\_init\_visgen
  , stan::gm::generate\_local\_var\_init\_nan\_visgen
  , stan::gm::init\_local\_var\_visgen
  , stan::gm::is\_nil\_op
  , stan::gm::is\_no\_op\_statement\_vis
  , stan::gm::left\_division\_expr
  , stan::gm::local\_var\_decl\_visgen
  , stan::gm::logical\_negate\_expr
  , stan::gm::member\_var\_decl\_visgen
  , stan::gm::modulus\_expr
  , stan::gm::multiplication\_expr
  , stan::gm::name\_vis
  , stan::gm::negate\_expr
  , stan::gm::printable\_visgen
  , stan::gm::remove\_loop\_identifier
  , stan::gm::returns\_type\_vis
  , stan::gm::scope\_lp
  , stan::gm::set\_allows\_sampling\_origin
  , stan::gm::set\_fun\_type2
  , stan::gm::set\_fun\_type3
  , stan::gm::set\_fun\_type
  , stan::gm::set\_fun\_type\_named
  , stan::gm::set\_param\_ranges\_visgen
  , stan::gm::set\_var\_type
  , stan::gm::set\_void\_function
  , stan::gm::statement\_visgen
  , stan::gm::subtraction\_expr3
  , stan::gm::transpose\_expr
  , stan::gm::unconstrained\_param\_names\_visgen
  , stan::gm::unscope\_locals
  , stan::gm::unscope\_variables
  , stan::gm::validate\_allow\_sample
  , stan::gm::validate\_assignment
  , stan::gm::validate\_declarations
  , stan::gm::validate\_expr\_type3
  , stan::gm::validate\_int\_expr2
  , stan::gm::validate\_int\_expr3
  , stan::gm::validate\_integrate\_ode
  , stan::gm::validate\_non\_void\_arg\_function
  , stan::gm::validate\_non\_void\_expression
  , stan::gm::validate\_return\_allowed
  , stan::gm::validate\_return\_type
  , stan::gm::validate\_sample
  , stan::gm::validate\_transformed\_params\_visgen
  , stan::gm::validate\_var\_decl\_visgen
  , stan::gm::validate\_void\_return\_allowed
  , stan::gm::var\_resizing\_visgen
  , stan::gm::var\_size\_validating\_visgen
  , stan::gm::write\_array\_vars\_visgen
  , stan::gm::write\_array\_visgen
  , stan::gm::write\_csv\_header\_visgen
  , stan::gm::write\_csv\_vars\_visgen
  , stan::gm::write\_csv\_visgen
  , stan::gm::write\_dims\_visgen
  , stan::gm::write\_param\_names\_visgen
  , stan::math::coupled\_ode\_observer
  , stan::math::coupled\_ode\_system< F, double, double >
  , stan::math::coupled\_ode\_system< F, double, stan::agrad::var >
  , stan::math::coupled\_ode\_system< F, stan::agrad::var, double >
  , stan::math::coupled\_ode\_system< F, stan::agrad::var, stan::agrad::var >
  , stan::model::model\_functional< M >
  , stan::optimization::ModelAdaptor< M >
  , validate\_decl\_constraints
  , validate\_double\_expr
  , validate\_identifier
  , validate\_int\_data\_expr
  , validate\_int\_expr
- operator\*()
  : stan::agrad::var
- operator\*=()
  : stan::agrad::fvar< T >
  , stan::agrad::var
  , stan::gm::expression
- operator++()
  : stan::agrad::fvar< T >
- operator+=()
  : stan::agrad::fvar< T >
  , stan::agrad::var
  , stan::gm::expression
- operator--()
  : stan::agrad::fvar< T >
- operator-=()
  : stan::agrad::fvar< T >
  , stan::agrad::var
  , stan::gm::expression
- operator->()
  : stan::agrad::var
- operator/=()
  : stan::agrad::fvar< T >
  , stan::agrad::var
  , stan::gm::expression
- operator<()
  : stan::gm::expr\_type
- operator<<
  : stan::agrad::fvar< T >
  , stan::agrad::var
  , stan::agrad::vari
- operator<=()
  : stan::gm::expr\_type
- operator=()
  : stan::gm::array\_literal
  , stan::gm::double\_literal
  , stan::gm::int\_literal
  , stan::mcmc::ps\_point
- operator==()
  : stan::gm::expr\_type
- operator>()
  : stan::gm::expr\_type
- operator>=()
  : stan::gm::expr\_type
- operator[]()
  : stan::agrad::op\_matrix\_vari
  , stan::agrad::op\_vector\_vari
  , stan::DoubleVectorView< used, is\_vec >
  , stan::DoubleVectorView< true, false >
  , stan::DoubleVectorView< true, true >
  , stan::math::seq\_view< T, S >
  , stan::math::seq\_view< double, std::vector< int > >
  , stan::math::seq\_view< T, Eigen::Matrix< S, 1, Eigen::Dynamic > >
  , stan::math::seq\_view< T, Eigen::Matrix< S, Eigen::Dynamic, 1 > >
  , stan::math::seq\_view< T, Eigen::Matrix< S, Eigen::Dynamic, Eigen::Dynamic > >
  , stan::math::seq\_view< T, std::vector< S > >
  , stan::math::seq\_view< T, std::vector< std::vector< T > > >
  , stan::math::seq\_view< T, std::vector< T > >
  , stan::VectorView< T, is\_array, throw\_if\_accessed >
  , stan::VectorView< const double, false, false >
  , stan::VectorView< const T, is\_array, throw\_if\_accessed >
  , stan::VectorViewMvt< T, is\_array, throw\_if\_accessed >
  , stan::VectorViewMvt< const T, is\_array, throw\_if\_accessed >
- opt\_dims\_r
  : stan::gm::statement\_grammar< Iterator >
  , stan::gm::var\_decls\_grammar< Iterator >
- ordered()
  : stan::io::reader< T >
- ordered\_constrain()
  : stan::io::reader< T >
- ordered\_decl\_r
  : stan::gm::var\_decls\_grammar< Iterator >
- ordered\_unconstrain()
  : stan::io::writer< T >
- ordered\_var\_decl()
  : stan::gm::ordered\_var\_decl
- out\_stream\_
  : stan::mcmc::base\_integrator< H, P >
  , stan::mcmc::base\_mcmc


---

[ Stan Home Page ]

*© 2011–2014,
Stan Development Team.*
